# Human Induced Pluripotent Stem Cells from Basic Research to Potential Clinical Applications in Cancer

**DOI:** 10.1155/2013/430290

**Published:** 2013-10-28

**Authors:** Teresa de Souza Fernandez, Cecilia de Souza Fernandez, André Luiz Mencalha

**Affiliations:** ^1^National Cancer Institute (INCA), Bone Marrow Transplantation Center (CEMO), Laboratory Division, 20230-130 Rio de Janeiro, RJ, Brazil; ^2^Mathematics and Statistics Institute, Federal Fluminense University (UFF), 24020-140 Niterói, RJ, Brazil; ^3^State University of Rio de Janeiro (UERJ), Roberto Alcântara Gomes Biology Institute, Department of Biophysics and Biometrics, 20551-030 Rio de Janeiro, RJ, Brazil

## Abstract

The human induced pluripotent stem cells (hiPSCs) are derived from a direct reprogramming of human somatic cells to a pluripotent stage through ectopic expression of specific transcription factors. These cells have two important properties, which are the self-renewal capacity and the ability to differentiate into any cell type of the human body. So, the discovery of hiPSCs opens new opportunities in biomedical sciences, since these cells may be useful for understanding the mechanisms of diseases in the production of new diseases models, in drug development/drug toxicity tests, gene therapies, and cell replacement therapies. However, the hiPSCs technology has limitations including the potential for the development of genetic and epigenetic abnormalities leading to tumorigenicity. Nowadays, basic research in the hiPSCs field has made progress in the application of new strategies with the aim to enable an efficient production of high-quality of hiPSCs for safety and efficacy, necessary to the future application for clinical practice. In this review, we show the recent advances in hiPSCs' basic research and some potential clinical applications focusing on cancer. We also present the importance of the use of statistical methods to evaluate the possible validation for the hiPSCs for future therapeutic use toward personalized cell therapies.

## 1. Introduction

Cancer is a major cause of mortality through the world. This disease evolves by a process of clonal expansion, genetic diversification, and clonal selection. The dynamics are complex and with highly variable patterns of genetic diversity and resultant clonal architecture [1]. Cancer cells have diverse biological capabilities that are conferred by numerous genetic and epigenetic modifications [2]. Several studies have been done with the aim of identifying biomarkers involving cancer for the development of new molecular target therapies. In recent years, different high-throughput platforms have been used for the genomic, transcriptomic, proteomic, and epigenomic analyses to search for new biomarkers involved in cancer and to bring new insights into the several aspects of cancer pathophysiology including angiogenesis, immune evasion, metastasis, altered cell growth, death, and metabolism [2–7].

 There are several pioneering examples of genomic aberrations being discovered in cancer cells and the findings being successfully translated into therapeutic agents with considerable effects on the practice of cancer medicine. The first genomic alteration found to be consistently associated with a human malignancy, the chronic myeloid leukemia (CML), was the Philadelphia chromosome, discovery by Nowell and Hungerford in 1960 [8]. The cytogenetic and molecular studies showed that this chromosomal alteration involves a reciprocal translocation between chromosomes 9 and 22, resulting in a fusion gene, the BCR-ABL. The BCR-ABL fusion gene encodes a constitutively active leukemogenic protein tyrosine kinase [9]. More than 30 years after the discovery of the Philadelphia chromosome, a small molecule inhibitor of this CML biomarker was developed, the imatinib mesylate. BCR-ABL kinase activity is inhibited by the selective activity of imatinib, a target agent that has demonstrated remarkable efficacy and tolerability. This is the first example of a target molecular therapeutic agent in cancer [10, 11]. It has been shown that imatinib blocks the cells proliferation and induces apoptosis in BCR-ABL expressing hematopoietic cells. Imatinib has been used as a first line therapy for CML patients. Different patterns of response to imatinib treatment have been recognized, ranging from best-case scenarios of rapid and unwavering response to difficult situations of intolerance and resistance, with the appearance of clonal cytogenetic abnormalities in Philadelphia chromosome-negative cells [12–14]. The resistant cancer cells emerged in different kinds of tumors, and research groups are studying these molecular mechanisms, especially in cancer stem cells (CSC) because of their dual role, as a tumor-initiating cell and as a source of treatment resistance cells [15–18].

Several approaches have been used to understand cancer pathogenesis, as animal models and cell cultures, using mainly the cell lines. Much of our understanding of cancer cell biology, including the aspects of gene regulation and signaling pathways, has come from studies of cancer cells in culture. But, theoretically, the best model to study cancer is the primary patient samples, but the amount of obtained cells may be inadequate for various analyses [2, 19, 20]. So, the recent discovery of the human induced pluripotent stem cells, hiPSCs, opens a new perspective to study the biology of different diseases, including cancer [19–21]. The hiPSCs are being used to make disease models, to develop new drugs, to test toxicity, and in regenerative medicine. The reprogramming technology offers the potential to treat many diseases, including neurodegenerative diseases, cardiovascular diseases, and diabetes. In theory, easily accessible cell types (such as skin fibroblasts) could be obtained from a patient and reprogrammed, effectively recapitulating the patients' disease in a culture system. Such cells could then serve as the basis for autologous cell replacement. However, depending on the methods used, reprogramming adult cells to obtain hiPSCs may pose significant risks that could limit their use in clinical practice. For example, if viruses are used to genomically alter the cells, the expression of cancer-causing genes “oncogenes” may potentially be triggered. So, many different groups have successfully generated iPSCs, but due to different techniques, their methods of calculating efficiency of conversion are varied [22–24]. In this review, we show the recent advances in hiPSCs basic research and some potential clinical applications focusing on cancer. We also present the importance of the use of statistical methods to evaluate the possible validation for the hiPSCs for future therapeutic use toward personalized cell therapies.

## 2. Human Induced Pluripotent Stem Cells: Discovery and the Development of Different Methods to Generate hiPSCs

The first generated induced pluripotent stem cells (iPSCs) were in mice by the Yamanaka's group at Kyoto University, Japan, in 2006. It is a recent discovery that iPSCs are derived from somatic cells through ectopic expression of specific transcription factors [25]. In 2007, human iPSCs were first generated by the same group by transducing adult human dermal fibroblasts with viral vectors carrying the key pluripotency genes, Oct3/4, Sox2, Klf4, and c-Myc (Yamanaka factors), using a retroviral system [26]. In 2007, Thomson's group at the University of Wisconsin-Madison, EUA, also generated human iPSCs. They used the factors Oct4, Sox2, Nanog, and LIN28 and a lentiviral system to reprogram human somatic cells to pluripotent stem cells that exhibit the essential characteristics of embryonic stem cells (ESCs) [27]. The ESCs are pluripotent cells derived from the inner cell mass of the preimplantation blastocyst. These cells are potential renewable sources of all human tissues for regenerative medicine, and, for this reason, they are very valuable to understand the early events of human development, in gene therapy and for new drug discovery. However, the usage of ESCs is a highly controversial issue on moral, social, and ethical grounds. This is because the process involves the destruction of a blastocyst, which is considered a human embryo with the potential of developing into a person. The research using the ESCs is prohibited in some countries, while in other countries the research using the ESCs is allowed under legislation but remains tightly restricted [22]. So, the research using hiPSCs, which are derived from human somatic cells, does not present the ethical dilemmas as the research using the ESCs. In Table 1, we show the advantages and disadvantages in using the iPSCs.

In the experiment of Dr. Yamanaka, the ectopic expression of “embryonic factors” was cloned and promoted in the differentiated human cells. Initially, 24 genes were analyzed and selected. Among these genes, there were genes involved in the maintenance of pluripotency like Oct3/4, Nanog, and Sox2; there were genes overexpressed in the tumors related with fast proliferation and maintenance of undifferentiated stage like STAT-3, Ras, c-Myc, Klf4, and Beta-catenin and genes expressed in the early stages of development such as FGF4, Zfp296, Utf1, and others. For the expression of these genes, they were selectively amplified from cDNA template by PCR, cloned into plasmid and *in vitro* introduced in fibroblast cells through retroviral transduction. After infections and confirmation of expression of the introduced genes, the fibroblast cells were analyzed to observe cell phenotype. Dr. Yamanaka performed a series of evaluations in a single or combined gene to verify which ones were essential or able to induce alterations in the differentiated fibroblast cell morphology, growth, and gene expression profile similar to ESCs. Among the initial genes studied, only the Oct3/4, Sox2, c-Myc, and Klf4 appeared to be important, generating the iPSCs. This study established a new concept in the science scenario: the *in vitro* induced pluripotent stem cells. The hiPSCs technology represents an important platform with the potential to advance in medical therapy by personalizing regenerative medicine and by creating new human disease models for research and therapeutic tests. The discovery that adult somatic stem cells can be reprogrammed into pluripotent cells is so important that, in 2012, Dr. Yamanaka was awarded with the Nobel Prize in Physiology or Medicine [25, 26, 28].

Basically, the methodology used to generate hiPSCs implies in the specific gene amplification by PCR, insertion of this product in a DNA vector, and introduction of this cloned gene in the host cell. The foreign DNA vector can be inserted in the receptor by several different ways, like the viral transduction. The method using viral transduction has efficiency to introduce the DNA vector inside cell and successful integrating of the DNA cloned in the host cell's genome, and this is the main advantage of viral method. The DNA vector viral integrates in host genome cell particularly due to long terminal repeats (LTR) present in both extremities of virus genome. These LTRs are compound by hundreds of nucleotides repetitions that, by recombination, attach the DNA inner contained in genomes [29]. 

Many approaches have used viral particles carrying DNA constructions that can be integrated in the genome's cell randomly. In fact, it is the main counterpart of iPSCs utilization. Therefore, reprogramming by cloning with the usage of viral strategies and long-term culture can also induce abnormalities in these pluripotent cells. *In vitro* cultures, sometimes iPSCs have demonstrated genomic instability. Unlike other stem cell cultures, the genomic instability is more common in early passages [30]. It is believed that this phenomenon is due to genetic reprogramming [30, 31]. This enhanced genomic instability in iPSCs can involve p53 protein inactivation, which is important to proliferation and DNA repair machinery activation in response to DNA damage [32]. 

Additionally, the viral DNA that carries cloned gene of interest can integrate in any *loci* in genome host cells. This implies many consequences, such as (1) integration into DNA sequence that encodes essential gene, disrupting its function which can cause loss of cell viability; (2) disrupt regions that coordinate expression of important genes, like promoter or enhancers regions, mainly if these genes contain “tumor suppression functions”; (3) the viral DNA may integrate in DNA regions that are responsible for negative regulation of “oncogenes,” allowing their constitutive expressions [33, 34]. Chromosomal instability, mutational possibilities, and use of known oncogenes, c-Myc and Klf4, to produce iPSCs, have implicated in the high incidence of cancer development in preclinical tests induced by iPSCs [35]. These observations have increased the discussion about the possibility of the usage of iPSCs in cellular therapies.

Another point is that the stimulation of loss of differentiation state to generate iPSCs also involves epigenetics reprogramming process and differential expression of noncoding functional RNA (ncRNA). A recent study discovered that there are more miRNA upregulated in the iPSCs than in the ESCs. These miRNAs have been frequently found related in the cancer development [36]. 

Most strategies currently under use to generate iPSCs are based on gene delivery via retroviral or lentiviral vectors [26, 27, 37–39]. However, most experiments involved integration in the host cell genome with an identified risk for insertional mutagenesis and oncogenic transformation. To avoid such risks, which are incompatible with therapeutic prospects, significant progress has been made with transgene-free reprogramming methods based, for example, on Sendai virus, direct mRNA, or protein delivery to achieve conversion of adult cells into hiPSCs [40–45]. So, there have been several improvements of the gene transduction method for making safe iPSCs. Due to an intense discussion about the use of hiPSCs in cellular therapies, since they are not completely safe, a lot of works, trying to establish *in vitro* stem cells derived from a variety of sources, has emerged. For example, bone marrow derived hematopoietic stem cells, multipotent mesenchymal stromal cells derived from bone marrow, umbilical cord blood, and adipose tissue. The ideal source of the cell to be isolated from the patients and used for reprogramming must have easy accessibility. This means that it is not necessary to have surgery to get the cells, it is possible to obtain them from a skin biopsy, for example, with minimal risk procedures, availability in large quantities, relatively high reprogramming efficiency, and fast iPSCs derivation speed [45]. Thus, new sources to obtain stem cell has also emerged; new strategies to induce cell reprogramming without the use of viral particles have been used aiming for safety and efficiency to generate hiPSCs with the purpose of their use in clinical practice [46–51]. For detection of high-quality hiPSCs, specialized cell tests may be conducted for making efficient differentiation protocols [52]. Now, basic research should be focused on characterizing the hiPSCs at cytogenetic and molecular levels to observe if these cells retain the genetic stability. It is necessary to understand how the cellular reprogramming works at molecular level, generating new knowledge in cell signaling pathways, comparing the different cell sources and the different methods used to generate the hiPSCs with the basic requirements of high quality and safety for their use in patients. In Table 2, a summary of the main methods used to generate iPSCs is shown.

## 3. Human iPSCs: Potential Clinical Applications in Cancer

This is the first review study focusing on the potential use of hiPSCs in clinical applications for cancer. We ask the following question: how can the hiPSCs, which may cause malignant transformation, be used for study and for possible application in the treatment of cancer? 

The hiPSCs can lead to clinical applications as the study of the disease biology, making disease models, developing new drugs, and testing toxicity. Recent progress in the reprogramming field has demonstrated important disease models using iPSCs in both gene target therapies, for example, the sickle cell anemia and augmentation therapy, for example, for Hemophilia A. The gene therapy refers to the introduction of genetic material into particular cells or tissues for therapeutic purposes especially in gene corrections for mutations in monogenic genetic diseases [22, 53].

Cancer is a complex disease, characterized by genetic and epigenetic alterations. Many researchers are trying to identify biomarkers involved in tumor initiation as well as the steps involved during the evolution of disease. The main purpose of using biomarkers is to develop new drugs for cancer therapy. Furthermore, the identification of biomarkers can be used for early diagnosis and for therapeutic stratification groups aiding the medical staff to choose the appropriate treatment for that patient [2, 54].

Theoretically, the best model to study cancer pathogenesis is the primary patient samples, but the amount of obtained cells may be inadequate for various analyses. Recently, it was reported that iPSCs can be generated not only from normal tissue cells but also from malignant cells [19, 55–58]. So, the hiPSCs are highly relevant to study the multiple stages of oncogenesis, from the initial cellular transformation to the hierarchical organization of established malignancies providing a human cell model to study the stages of disease [59, 60]. In this sense, there are some examples. Kim and colleagues (2013) used the hiPSCs as a model to study the pancreatic ductal adenocarcinoma (PDAC). This cancer carries a dismal prognosis and lack a human cell model of early disease progression. In this study, the authors made the following hypothesis: if human PADC cells were converted to pluripotency and then allowed to differentiate back into pancreatic tissue, they might undergo early stages of cancer [59]. So, the iPSCs technology can provide a live human cell model of early pancreatic cancer and disease progression.

Another example for the potential clinical applications of hiPSCs in disease modeling for studying cancer is in hematological malignancies. Primary samples of hematologic malignancy are usually difficult to be expanded in cultures. However, after they are reprogrammed to iPSCs, they can expand unlimitedly. The iPSCs technology has been used to study myeloproliferative diseases as chronic myeloid leukemia (CML) [19] and juvenile myelomonocytic leukemia (JMML) [61]. Many studies are being performed to elucidate the mechanisms of tyrosine kinase inhibitor (TKI) resistance in CML stem cells and to overcome the resistance in these patients. Kumano and colleagues (2012) established the CML-iPSCs by Sendai virus system and confirmed the resistance of these cells to imatinib [19]. So, they developed a model to study the CML disease and the TKI resistance. Another example for the use of iPSCs is the JMML. JMML is an aggressive myeloproliferative neoplasm of young children initiated by mutations that deregulated cytokine receptor signaling. Children with this disease have a poor prognosis. Gandre-Babbe [61] generated iPSCs from two JMML patients. In this study, the authors suggested the relevance of this method to explore the pathophysiology and treatment of JMML [61]. Emerging developments of iPSCs research can be used as a tool in modeling hematopoietic disorders and could lead to new clinical applications in gene and cell therapies [20]. The advantage of using disease modeling with iPSCs technology is that it allows the generation of pluripotent cells from any individual in the context of his/her own particular genetic identity including individuals with simple forms of disease and those with complex multifactorial diseases of unknown genetic identity [45]. In drug screening, the use of hiPSCs would be used to verify the response to a specific target gene and to research the single nucleotide polymorphism related to each individual that influences the ability of an individual to effectively metabolize and clear drugs and toxins. In particular, hepatotoxicity and cardiotoxicity are two principal causes of drug failure during preclinical testing. The variability in individual responses to potential therapeutic agents is also a major problem in effective drug development. The advantage of iPSCs technology is that it allows the generation of various cell lines that may represent the genetic and potentially epigenetic variation of a broad spectrum of the population. This approach used the *in vitro* model of disease to identify new drugs to treat disease [45].

Although some studies showed that cancer-derived hiPSCs is possible (Table 3), it is necessary a continuous progress in the iPSCs technology. Reprogramming cancer cells has been demonstrated to be harder than generation of normal iPSCs because of the genetic and epigenetic status of these cells. To try to overcome this difficulty, some researchers are testing other possibilities to generate cancer-derived hiPSCs by the application of other factors in addition to the Yamanaka factors, such as exogenous expression of miRNA302 and chemical compounds, as azacitidine (DNA methyltransferase inhibitor) and knockdown of p53, p21, and Ink4/Arf [19, 62]. Another point, here, for the normal and cancer cells, it is the genes delivery systems for the iPSCs generation. The integration site of retrovirus in the iPSCs may affect the gene expression and change the disease phenotype after redifferentiating them into the original lineages. So, efficient induction of transgene-free iPSCs such as using Sendai virus and episomal systems has been reported [19, 48, 57]. But, we can have in mind, as mentioned by Ramos-Mejia and collaborators (2012), that the difficulties in reprogramming cancer cells do not seem exclusively due to technical barriers or the need for improved reprogramming technologies. But, it seems that the biological barriers such as cancer-specific genetic mutations, epigenetic remodeling, or accumulation of DNA damage may influence the reprogramming of human cancer cells [63].

The cancer-derived hiPSCs represents important systems for modeling cancer pathogenesis, aiding in the discovery of new diagnostic and prognostic biomarkers, and for the development of new therapies for cancer. For example, Yang and collaborators (2012) demonstrated a tumor tropism of intravenously injected human iPSC-derived neural stem cells and their gene therapy application in a metastatic breast cancer mouse model. In this study, the authors used a lentiviral transduction method to derive hiPSCs from primary human fibroblasts and then generated neural stem cells (NSCs) from the iPSCs. The NSCs are able to home not only on brain tumors but also on solid tumors of a nonneural origin. This intrinsic tropism occurs because the presence of cytokines, chemokines, and growth factors released from the tumor cells. Yang and collaborators investigated whether the iPSCs derived NCS can be used as a cellular delivery vehicle for cancer gene therapy. For this propose, the cells were transduced with a baculoviral vector containing the herpes simplex virus thymidine kinase suicide gene and injected through tail vein into tumor-bearing mice. The transduced NCSs were effective in inhibiting the growth of the breast tumor and the metastatic spread of the cancer cells in the presence of ganciclovir, leading to the prolonged survival of the tumor-bearing mice. This study demonstrated the use of iPSC-derived NSCs for cancer gene therapy [64].

A potential clinical application of hiPSCs in cancer is in the field of immunotherapy [66–69]. Traditional treatment modalities are all based on destroying cancer cell by irradiation, chemotherapy, or surgery. Although, they can effectively kill or remove cancer cells, the use of these treatments often is limited because a number of health cells also tend to be destroyed and, in some cases, may occur the recidive of cancer. In the case of cancer, the immune system alone often fails to effectively fight the tumor for the following reasons: (1) the normal immune system is “blind” to tumor cells because the tumor cells are derived from the body's own cells. The body “thinks” about the tumor as “self,” a phenomenon known as tumor tolerance; (2) the immune system may recognize certain cancer cells, but the response may not be strong enough to destroy cancer; (3) the tumor has the ability to defend itself secreting some substances that allow its survival and expansion. In the case of cancer, the immune system needs a boost to enhance its response to become more effective. So, the immunotherapy strategies include antitumor monoclonal antibodies, cancer vaccines, adoptive transfer of *ex vivo* activated T or natural killer cells, and administration of antibodies that either stimulate immune cells or block immune inhibitory pathways. The impact of immunotherapy was initially demonstrated in patients with advanced cancer and then translated to the adjuvant setting of patients with operable disease at high risk for postoperative recurrence [70]. 

Therapies based on the use of autologous immune cells are among the best candidates for cancer immunotherapy. The dendritic cell vaccines have demonstrated very encouraging responses for some solid tumors, while in melanoma T-cell therapies have exceeded 70% objective response rates in selected Phase I trial [71]. However, it is difficult to obtain a sufficient number of functional dendritic cells (DCs) in DC-based immunotherapy. In this sense, some studies are being performed using the iPSCs. Iwamoto and colleagues (2013) used the iPS cell-derived DCs (iPSDCs) and compared the therapeutic efficacy of iPSDCs and the equivalent to that of bone marrow-derived DCs (BMDCs). In this study, the authors examined the capacity for maturation of iPSDCs compared with that of BMDCs in addition to the capacity for migration of iPSDCs to regional lymph nodes. The therapeutic efficacy of the vaccination was examined in a subcutaneous tumor model. The vaccination with genetically modified iPSDCs achieved a level of therapeutic efficacy as high as vaccination with BMDCs. This study showed experimentally that genetically modified iPSDCs have an equal capacity of BMDCs in terms of tumor-associated antigen-specific therapeutic antitumor immunity. Therefore, vaccination strategy may be useful for future clinical application as a cancer vaccine [67].

The immunotherapy based on the adoptive transfer or gene-engineered T cells can mediate tumor regression in patients with metastatic cancer [72]. Adoptive T-cell immune therapy is based on the isolation of tumor-specific T cells from a cancer patient, *in vitro* activation, expansion of these T cells, and reinfusion of the T cells to the patient [73]. The adoptive immunotherapy with T cells is an effective therapeutic strategy for combating many types of cancer. However, the limitations associated with the number of antigen-specific T cells represent a major challenge to this approach [74]. The recent iPSCs technology and the development of an *in vitro* system for gene delivery are able to generate iPSCs from patients. The iPSCs have a great potential to be used in adoptive cell transfer of antigen-specific CD8(+) cytotoxic T lymphocytes [75, 76]. Some research groups are studying methods to generate T lymphocytes from iPSCs *in vitro* and *in vivo* programming antigen-specific T cells from iPSCs for promoting cancer immune surveillance [76]. 

Natural killer (NK) cells play a critical role in host immunity against cancer. In response, cancer develops mechanisms to escape NK cell attack or induce defective NK cells. Current NK cell-based cancer immunotherapy aims to overcome NK cell paralysis using several approaches. One approach is the genetic modification of fresh NK cells or NK cell lines to highly express cytokines, Fc receptors, and/or chimeric tumor-antigen receptors. Therapeutic NK cells can be derived from various sources, including peripheral or cord blood cells, stem cells, or even induced pluripotent stem cells (iPSCs), and a variety of stimulators can be used for large scale production in laboratories or good manufacturing practice [77].

Adult stem cell therapies have provided success for more than 50 years, through reconstitution of the hematopoietic system using bone marrow, umbilical cord blood, and mobilized peripheral blood transplantation. Mesenchymal stem cell (MSC) mediated therapy is a fast-growing field that has shown to be safe and effective in the treatment of various degenerative diseases and tissues injuries. The expansion and manipulation of human MSCs are important approaches to immune regulatory and regenerative cell therapies. MSCs are fibroblast-like cells of the BM microenvironment called “marrow stromal cells,” which were able to support hematopoiesis. These cells have adult stem cell properties as they could differentiate into cartilage, bone, adipocytes, and muscle cells. MSCs are a promising tool for cell therapies because they are easily accessible from various tissue sources as bone marrow (BM-MSC), fat, and umbilical cord [78]. These cells have been widely tested and showed efficacious in preclinical and clinical studies for cardiovascular and neurodegenerative diseases, orthopedic injuries, graft-versus-host disease (GvHD) following bone marrow transplantation, autoimmune diseases, and liver diseases [78, 79].

**Figure 1:**
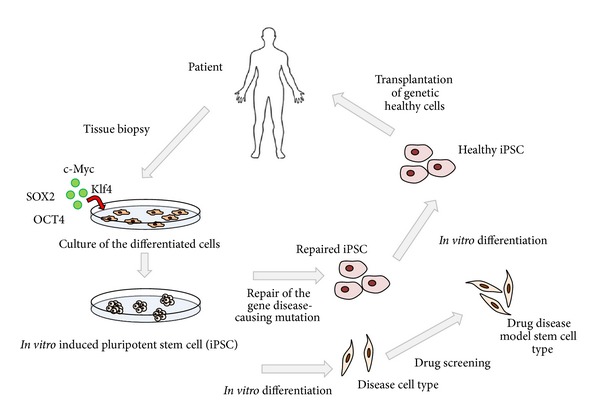
Potential applications of human iPSCs. The iPSCs technology can be potentially used in disease modeling, drug discovery, gene therapy, and cell replacement therapy. Differentiated cells are acquired by biopsies from human tissues and *in vitro* cultured under stem cell transcription factors, such as SOX2 (*SRY-box containing gene 2*), c-Myc (*v-myc avian myelocytomatosis viral oncogene homolog*), OCT4 (*octamer-binding transcription factor 3*), and KlF4 (*Kruppel-like factor 4*). After induction of pluripotency phenotype, the cells, known as iPSCs, can be utilized to redifferentiation in specific disease, to drug screening, or to have the genomic defect corrected, and then the iPSCs become able to be reutilized as health cells in the regenerative therapies.

Because BM-MSC can be easily harvested from adult sources and cultured *in vitro*, many preclinical and clinical studies have used BM-MSC. Although these cells show great potential for clinical use, there are some problems. The need for extensive cell number for use poses a risk of accumulating genetic and epigenetic abnormalities that could lead to malignant cell transformation. Binato and colleagues (2013) studied the stability of human MSCs during *in vitro *culture in several passages using cytogenetic, cellular, and molecular methods, and it was observed that these cells demonstrated chromosomal instability and molecular changes during passage 5 [80]. Although easy access to BM-MSC is recognized as a great advantage, extended *in vitro* cultures reduce the differentiation potential of MSC, which limits their therapeutic efficacy [78]. So, to overcome this problem, MSCs derived from iPSC may be considered for human cell and gene therapy applications as iPSCs have the potential to be expanded indefinitely without senescence. A greater regenerative potential of MSCs is observed derived from iPSCs which may be attributed to superior survival and engraftment after transplantation, because of higher telomerase activity and less senescence as compared to BM-MSC. Genetically manipulated MSCs may also serve as cellular therapeutics since MSCs can be used as a target drug delivery vehicles [78]. In Figure 1, we can see the generating of hiPSCs and the potential applications of these cells.

## 4. Statistical Methods to Evaluate the Possible Validation for the hiPSCs for Future Therapeutic Use

Medicine is full of mysteries. For centuries, people are trying to understand how the human body works. Many advances were made. As a consequence, human being has been living more and better.

The human body is a complex structure, influenced by many factors. So, it is difficult to answer medical questions. A tool that can help to ask such questions is based on a mathematical concept: the concept of probability. In fact, the tool we are talking about is the theory of mathematical statistics, which is the study of how to deal with data by means of probability models.

Clinical research relies on quantitative measurements. Impressions, intuitions, and beliefs are important in medicine, but only when they are together with a solid base of numerical information. This base allows more precise communication between clinicians and between clinicians and patients, as well as an error estimate. Clinical outcomes such as the occurrence of disease, death, symptoms, or functional impairment can be counted and expressed as numbers. In most clinical situations, diagnosis, prognosis, and treatment results are uncertain for an individual patient. A person will experiment a clinical outcome or not: the prediction is rarely accurate. Therefore, the prediction needs to be expressed as a probability. This can explain why probability models are important to clinical research.

Probability models lead to *statistical hypothesis tests* and *estimates*. They are used to draw inferences and reach conclusions about data, when only a part of a population, a sample, has been studied. When we consider a sample, we need to have in mind what variables we are considering under study. Also, the number of its elements is very important. For example, if we are interested in estimating *one mean*, the Central Limit Theorem establishes that the sampling distribution of means will be approximately normal even when its population is not distributed normally, provided that the sample size is large. If *n* denotes the number of elements of a sample, *n* ≥ 30 is our definition of “large” [81].

If we decide to approximate clinical measurements by a normal curve, we are deciding to use a parametric test. Because normal distribution has nice mathematical properties (bell-shaped, symmetric, etc.), using a parametric test leads to better results compared with a nonparametric one. In other words, we say that nonparametric tests are less powerful, in the sense that they lead to a small probability to reject the null hypothesis, when it is false.

The iPSCs are undifferentiated cells that have the capacity to proliferate in undifferentiated cells both *in vitro* and *in vivo* (self-renewal) and to differentiate into mature specialized cells. Because this is a new discovery, there are open questions regarding, mainly, the safe application of stem cell therapy using the iPSCs. As we have presented in this work, many different groups have successfully generated iPSCs, but due to different techniques, until now, there is no standard information about the safety and effectiveness of the use of iPSCs in the clinical practice.

**Figure 2:**
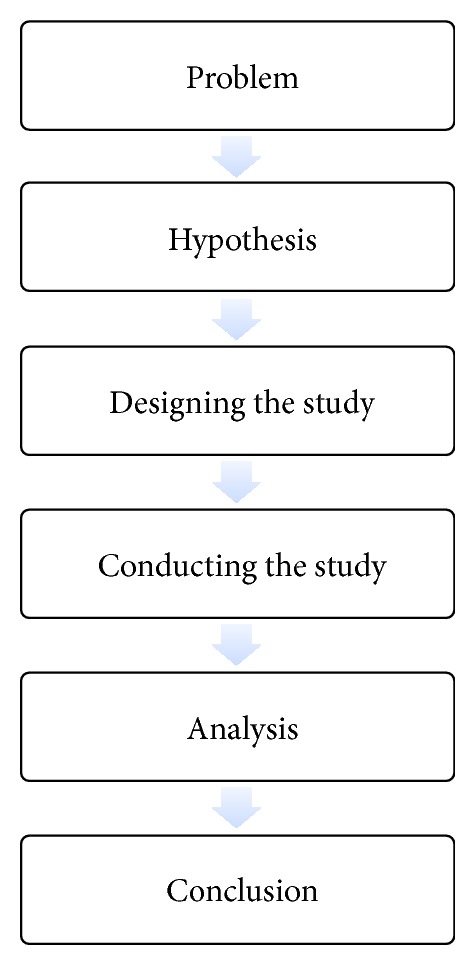
Structure of a clinical study.

All scientific studies aim to answer a question that arises by observations of the researcher or the results of previous studies. Structuring a study helps answering questions in a systematic way (Figure 2). We note that a question well formulated is of great importance to the success of a study.

In order to have a better understanding of how we can minimize the problems, which occurs with the use of iPSCs, we think it is important to consider the following questions.

1. How does the cellular reprogramming work at cytogenetic level?
2. How does the cellular reprogramming work at molecular level?
3. Is there an association between cell sources (fibroblasts from skin, stem cells from bone marrow, umbilical cord blood, and adipose tissue) and the self-renewal capacity?
4. Is there an association between sex of the patients and self-renewal capacity?
5. Do pediatric patients have more success than adult patients in the reprogramming therapy?
6. What kind of tissues can make the introduction of the hiPSCs easier?
7. Which methods used to generate the hiPSCs are better related with safety?
8. How can we compare different diseases and the use of the iPSCs?

There are several tests commonly used in the medical literature; they are presented in Table 4. When we use such tests, we compute a *P* value. The *P* value is the probability of obtaining a result as extreme as or more extreme than the sample value, assuming that the null hypothesis is true. The sample value is calculated. Depending on the test we use, there is a specific formula to calculate the sample value. Appropriate computer software can do such a calculation.

In many situations, populations are so large that it is impossible to describe their central tendency and dispersion by studying 100% of their members, or by studying a sufficiently large portion of population to justify treating sample statistics as population parameters. In other situations, clinicians may study a new phenomenon with little basis to determine a population parameter. In these cases, we use estimates. Two types of estimates of a population parameter can be used: a point estimate and an interval estimate. A point estimate is a single numerical value of a sample statistic used to estimate the corresponding population parameter. Point estimates are not used widely because, in general, values of some statistic can vary from sample to sample. So, an interval estimate is typically used. Interval estimates are also called confidence intervals. Confidence intervals provide more information on, for example, the mean of a variable in the considered population than just the sample mean. When the sample mean is calculated, we know that there is a sample to sample variation, that is, if another sample was selected (i.e., if other patients were selected), the sample mean would rarely be the same. Thus, the confidence interval provides a set built in such a way that if a large number of different samples were selected and we built confidence intervals for all of them, the value of the population mean would be contained in 95% of the intervals. In this case, we have a 95% confidence interval. In general, researchers use 95% confidence intervals, but 99% is also a very used.

We finish by saying that probability models are important tools that *can help* making decisions and must be used if the numerical outcomes are clinically meaningful. Accumulated experience and specific knowledge must be combined with numerical results to assess the usefulness of a medical decision.

## 5. Conclusions

An important point in the research using hiPSCs is that these cells do not present the ethical dilemmas as the research using the ESCs. Since the first description of iPSCs generation, there has been a great improvement in the methods to generate these cells. The main problem with using these cells is the possibility of developing tumors. However, basic research should aim at the improvement of methods to generate the iPSCs. It is also very important to obtain a characterization of these cells at cytogenetic and molecular levels, in order to understand how reprogramming works in signaling pathways. The different sources of cells to generate iPSCs may be compared. Many technical and basic knowledge are necessary before the use of iPSCs in the clinical practice. The possibility to induce pluripotency in somatic cells or even further to induce cell transdifferentiation through the forced expression of reprogramming factors has offered a new field for cancer research and future possible applications in the clinical practice. The recent findings regarding the use of iPSCs for modeling different types of cancer like solid tumors and hematological malignancies represent an ideal tool to study the multiple stages of cancer, for the discovery of new drugs designed for specific biomarkers and for testing drugs' toxicity. Another important point is the possibility to use the iPSCs for immunotherapy in cancer. So, the use of hiPSCs may contribute to the development of future personalized cell therapies and open new possibilities for the treatment of cancer patients. 

## Figures and Tables

**Table 1:** Advantages and disadvantages in iPSCs utilization.

Advantages	Disadvantages
Avoid human embryos' use	Oncogene use for induction iPSCs phenotype
Capacity to induce stem cell like phenotype	Use of integrative DNA methodology
New promises to cellular therapy	Genomic instability and aberrations
Possibility of studying several diseases, including cancer	Increase risk of the development of cancer

**Table 2:** Summary of the methods used to generate iPSCs.

Methodology	Cell type	Genome integration	Efficiency of iPSC induction	References
Retroviral transduction	Fibroblast, neuronal, keratinocyte, blood cells, adipose, and liver cells	Yes	High	Takahashi et al., 2007 [26]/Lowry et al., 2008 [37]
Lentiviral transduction	Fibroblast and keratinocyte	Yes	High	Yu et al., 2007 [27]/Moore, 2013 [38]
Inducible lentiviral transduction	Fibroblast, melanocytes, beta-cells, blood cells, and keratinocyte	Yes	High	Maherali et al., 2008 [39]
Adenoviral transduction	Fibroblast	No	Low	Stadtfeld et al., 2008 [40]
Plasmid vector	Fibroblast	No	Low	Si-Tayeb et al., 2010 [41]
Cell-free lysate or protein extract	Fibroblast and adipose stromal cells	No	Low	Kim et al., 2009 [42]
Cellfusion	Fibroblasts and adult thymocytes	No	Low	Cowan et al., 2005 [43]
Sendai viral transduction	Fibroblast and CD34^+^ cord blood cells/CD34^+^ cells from CML patient/ Peripheral blood mononuclear cells	No	High	Ban et al., 2011 [49]/Kumano et al., 2012 [19]/Churko et al., 2013 [50]
Minicircle DNA	Adipose stem cells	No	High	Narsinh et al., 2011 [51]
Episomal vectors	Mononuclear bone marrow and cord blood cells	No	High	Hu and Slukvin, 2013 [48]

**Table 3:** Summary of cancer-derived hiPSCs.

Type of cancer (hematologic malignancies and solid tumors)	Aim of study	Method of generation of the cancer hiPSCs	References
Myeloproliferative disorder (MPD) with JAK2-V617F somatic mutation	To generate iPS cells to provide a renewable cell source and a prospective hematopoiesis model for investigating MPD pathogenesis	Frozen peripheral blood CD34^+^ cells from 2 patients with MPD/retroviral transduction	Ye et al., 2009 [55]
Chronic myeloid leukemia (CML)	To address whether human cancer cells can be reprogrammed into iPSCs	Cell line, KBM7, derived from blast crisis stage of CML/retroviral transduction	Carette et al., 2010 [56]
Chronic myeloid leukemia (CML)	To eliminate the genomic integration and background transgene expression, toward advancing iPSCs technology for the modeling of blood diseases	Bone marrow mononuclear cells from a patient with CML (chronic phase)/episomal vectors	Hu et al., 2011 [57]
Chronic myeloid leukemia (CML)	To investigate CML pathogenesis on the basis of patient-derived samples	Two patients samples of CML (chronic phase) bone marrow cells, retrovirus and Sendai virus system	Kumano et al., 2012 [19]
Juvenile myelomonocytic leukemia (JMML)	To explore the pathophysiology and treatment of JMML	Two pediatric patient's samples from bone marrow or peripheral blood/lentivirus	Gandre-Babbe et al., 2013 [61]
Gastrointestinal cancer	To study new cancer therapies via reprogramming approaches in cancer cells	Gastrointestinal cell lines of cancers from esophageal, stomach, colorectal, pancreas, and liver and bile ducts/lentiviral and retroviral	Miyoshi et al., 2010 [58]
Gastrointestinal cancer	Generate a human cell model of early pancreatic cancer and disease progression for biomarkers detection for useful diagnosis	Tissue from the center of pancreatic ductal adenocarcinoma (PDAC) sample of patient/lentivirus system	Kim et al., 2013 [59]

**Table 4:** Statistical tests usually used in the medical literature.

To test the statistical significance of the difference between...		
Two or more proportions	Chi-square	Nonparametric
Two proportions	Fisher's exact	Parametric
Two medians	Mann-Whitney	Nonparametric
Two means	*t*-Student	Parametric
More than two means	Kruskal-Wallis (one-factor)	Nonparametric
Two or more than two variances	Bartlett	Parametric
More than two means	ANOVA (one-factor)	Parametric
More than two means	ANOVA (more-factors)	Parametric
To test the correlation between two variables	Spearman's rank correlation test	Nonparametric
To test the correlation between two variables	Pearson's correlation test	Parametric

Adapted from: Fernandez et al. 2012 [65].
